# Nominal group consensus process to determine Association of Surgeons in Training quality indicators for integrated clinical academic surgical training across the UK

**DOI:** 10.1093/bjsopen/zrac048

**Published:** 2022-04-20

**Authors:** Association of Surgeons in Training (ASiT), J. Glasbey, N. Blencowe, J. Barclay, A. J. Beamish, S. Bhatia, W. Bolton, C. Brennan, S. J. Chapman J, M. Clements, E. Elsey, R. Fish, V. J. Gokani, G. Humm, K. Hurst, P. Hutchinson, C. Jones, V. Kasivisvanathan, N. Keates, S. Lawday, M. J. Lee, C. Lovegrove, H. Mann, J. Mason, F. McDermott, K. McLean, H. Mohan, D. Nally, P. Pucher, S. Staight, A. K. Sorial, I. Trout, J. Burke

*Dear Editor*

In the UK, a unique integrated clinical academic training pathway exists to support co-development of clinical skills with skills in research, teaching, and leadership for trainees with an academic interest^1,2^. In surgery, as a craft specialty, the balance of acquisition and maintenance of technical skills combined with academic development poses a challenge to surgical trainees and programme directors. As a result of this, clinical academic trainees in surgery have reported that their dedicated academic time is commonly lost to clinical service commitments, and a lack of transparency around both clinical and academic expectations^3^. The aim of this study was to agree consensus quality indicators for integrated clinical academic training posts (AQIs) to allow benchmarking and improvement of academic surgical training in the UK. Details of background, research methods, and supplementary figures and tables are in the *Supplementary material*.

This development process followed a modified nominal group consensus methodology across five sequential stages: in-person scoping exercise; virtual stakeholder consultation; nominal group consensus meeting; virtual stakeholder feedback; and dissemination and implementation (*Supplementary material*). Design and reporting followed best practice methodology for consensus research^4,5^. Contributors were invited from the Association of Surgeons in Training (ASiT) mailing list, social media, and through the UK Clinical Academic Training Forum. The expertise matrix was designed to sample trainees of different sexes (male/female), training grades (postgraduate year 1–2/year 3–5/year 5 and above), and types (current integrated academic trainees/past integrated academic trainees/trainees with an academic interest). All AQIs were required to be examples of existing real-world practice in one or more UK region (deemed to be feasible not aspirational).

After stage 1 and 2, 16 candidate AQIs were drafted for inclusion in the nominal group meeting (*Supplementary material*). Thirteen nominal group members were present for the face-to-face stage 3 discussion, with a diverse range of experiences. Four new AQIs were identified during silent generation, and all 20 AQIs were discussed in the sharing ideas stage (*Supplementary material*). Wording was further refined for 7 indicators in stage 5, before final agreement of 20 AQIs across seven domains (*Table 1*): supervision and mentorship (assigned supervisor, clear objectives, academic programme director, and administrator), protected academic time (protected from clinical work, not used to fill rota gaps, trust responsibilities, flexibility to arrange time), clinical work (prioritized training, back to training interview, flexible on-call commitment), audit nd accountability (annual review with the quality indicators), financial assistance and resourcing (clinical and academic study budgets, local working space), training progression (clinical training plans, dedicated academic Annual Review of Competency Progression, flexible completion dates), and recruitment (involving the clinical programme director). The AQI indicators were designed to be delivered with existing resources (not to detract from resources available for non-integrated trainees). Key external stakeholder groups were identified and illustrative examples implementation of the AQIs were provided (*Supplementary material*).

**Table 1:** Final ASiT integrated clinical academic surgical training quality indicators

**Supervision and mentorship**	
AQI1	Academic trainees in surgery should have an assigned academic supervisor. Where possible this should match their specialty interest and trainees should be supported to change academic supervisors if their research interests do not match
AQI2	Academic supervisors should set clear objectives at the start of each training year in partnership with the academic trainees and these should be reviewed on at least a quarterly basis
AQI3	Academic trainees should have a dedicated academic training programme director whose responsibility it is to safeguard interests of the academic trainee and liaise with local trust clinicians/managers where required
AQI4	Academic trainees should have a named regional academic administrator who can provide information on academic training/educational opportunities and can liaise with local trusts
**Protected academic time**	
AQI5	The contracted academic time of academic trainees should be protected from non-academic activities. This should be communicated to the departmental NHS managers, rota coordinator, and local clinical supervisors by the training programme director at least 3 months before starting the placement, and any unmet service need covered by the trust
AQI6	Academic trainees should not be required to fill ‘rota gaps’ where these arise during their dedicated academic time, to allow for progression in line with a competency-based curriculum
AQI7	If academic trainees’ clinical duties are left uncovered because of protected academic time, the trust should be responsible for providing cover
AQI8	Academic trainees should be given flexibility to arrange their academic time in days/weeks/months, according to what frequency best suits their academic work
**Clinical work**	
AQI9	Academic trainees should have key local training opportunities prioritized, which may be at the expense of some service delivery
AQI10	Academic trainees that are on an on-call rota should be offered flexibility to reduce their on-call commitment to reflect their reduced overall clinical time (pro rata, akin to ‘Less-Than-Full-Time’ training)
AQI11	Following an extended interval of academic time, academic trainees should be able to request a ‘back to training’ interview and, where desired, keep-in-touch days, or phased return to clinical duties
**Audit and accountability**	
AQI12	Annual review of academic trainees’ experiences within training units should be undertaken by programme directors. This could be supported, for example, by the ASiT integrated clinical academic surgical training quality indicators
AQI13	Quality indicators for academic training posts should be clearly communicated to all hospital administrative and co-ordinating staff through which academic trainees rotate
**Financial assistance and resourcing**	
AQI14	Academic trainees in surgery should have access to the same clinical study budget for additional clinical training courses as non-academic trainees
AQI15	The process for academic trainees to access additional training and study budget available from the National Institute for Health Research should be transparent, and made available by the academic training programme director at the start of the integrated training post
AQI16	Academic trainees should have a local working space (such as a shared office), and institutional library access made available for them to complete academic work during clinical postings
**Training progression**	
AQI17	Academic trainees should have individualized clinical training plans discussed at ARCP, to provide flexibility, and understanding in placements given the need to balance academic and clinical commitments
AQI18	Academic trainees should have a dedicated academic ARCP (either as part of a clinical ARCP, or separately) where the panel specifically review their academic objectives and outcomes
AQI19	Academic trainees should have the flexibility to extend their CCT dates where required, reflecting their reduced clinical commitments. This decision should be made with the trainee, based on competency-based progression
**Recruitment**	
AQI20	Clinical training programme directors should be part of the selection panels for integrated academic clinical training posts, where possible

ASiT, Association of Surgeons in Training; ARCP, Annual Review of Competency Progression; CCT, Certificate of completion of training.

Finally, the group designed an annual audit to provide feedback to key external stakeholders. The audit will capture experience of integrated clinical academic trainees in surgery over a single training year. This seeks to have two main benefits. First, it will empower local integrated trainees with a set of agreed quality standards to discuss with their local training directors and hospital administrators (a ‘bottom up’ approach). Second, by leveraging real-world data, we hope to work with key external stakeholders to promote high-quality, inclusive, and enjoyable integrated academic clinical training (a ‘top down’ approach). The annual audit will launch in April 2022 and is available at: https://bit.ly/ASiTAQI.

## Collaborators

**Association of Surgeons in Training**

J. Glasbey, N. Blencowe, J. Barclay, A. J. Beamish, S. Bhatia, W. Bolton, C. Brennan, S. J. Chapman, J, M. Clements, E. Elsey, R. Fish, V. J. Gokani, G. Humm, K. Hurst, P. Hutchinson, C. Jones, V. Kasivisvanathan, N. Keates, S. Lawday, M. J. Lee, C. Lovegrove, H. Mann, J. Mason, F. McDermott, K. McLean, H. Mohan, D. Nally, P. Pucher, S. Staight, A. K. Sorial, I. Trout and J. Burke

## Supplementary Material

zrac048_Supplementary_Data
