# Supplementary material for: Nominal group consensus process to determine Association of Surgeons in Training quality indicators for integrated clinical academic surgical training across the UK
Source: BJS Open. 2022 Apr 20;6(2):zrac048. doi: 10.1093/bjsopen/zrac048 (PMC9019067; doi:10.1093/bjsopen/zrac048)

**Nominal group consensus process to determine ASIT quality indicators for integrated clinical academic surgical training across the United Kingdom**

**Appendix S1. Research abstract**

*Introduction*

The aim of this study was to agree consensus quality indicators for integrated clinical-academic surgical training programmes to allow benchmarking and improvement of academic surgical training in the UK.

*Methods*

This study followed a modified nominal group consensus methodology across five sequential stages: (1) in-person scoping exercise; (2) virtual stakeholder consultation; (3) nominal group consensus meeting; (4) virtual stakeholder feedback; (5) dissemination and implementation. Design and reporting followed best practice methodology for consensus research.

*Results*

A total of 27 trainees from both integrated clinical academic and non-integrated backgrounds were sampled in the consensus process. 20 candidate quality indicators were included in the round robin polling, with 30.0% (6/20) accepted with no change to wording and 70.0% (14/20) accepted with change to wording. The consensus indicators spanned 7 domains including supervision and mentorship (assigned supervisor, clear objectives, academic programme director and administrator), protected academic time (protected from clinical work, not used to fill rota gaps, trust responsibilities, flexibility to arrange time), clinical work (prioritised training, back to training interview, flexible on-call commitment), audit and accountability (annual review using the quality indicators), financial assistance and resourcing (clinical and academic study budgets, local working space), training progression (clinical training plans, dedicated academic ARCP, flexible completion dates), and recruitment (involving the clinical programme director).

*Conclusion*

The ASiT integrated clinical academic surgical training quality indicators aim to improve advocacy for high-quality and equitable experiences of academic training in the UK.

**Appendix S2. Extended background**

In the UK, a unique integrated clinical academic training pathway exists to support co-development of clinical skills with skills in research, teaching, and leadership for trainees with an academic interest^1^. Upon successful application, trainees spend between 25% and 75% of their time in a dedicated academic role funded either by the National Institute of Health Research (NIHR), or through local academic institutions. Research has demonstrated that this scheme has been successful in developing medical research leaders of the future, who are able to step across the translational clinical academic interface to ensure research is important and meaningful to the patients they treat ^2,3^.

In surgery, as a craft specialty, the balance of acquisition and maintenance of technical skills combined with academic development poses a challenge to surgical trainees and programme directors. As a result of this clinical academic trainees in surgery have reported that their dedicated academic time is commonly lost to clinical service commitments, and a lack of transparency around both clinical and academic expectations ^4^. Sadly, over 40% had also reported negative sentiment relating to their status as an academic trainee. This highlighted an urgent need for trainee organisations to collaborate with programme directors and policy makers to improve the status and support within academic surgical training.

The Joint Committee on Surgical Training (JCST) have an existing set of dedicated Quality Indicators (QIs) for each surgical training programme to which Local Education and Training Boards (or equivalent; being the organisation responsible for coordinate of surgical training posts in the UK) are held accountable; these include specific sessional activities, holistic development objectives and supervision requirements. However, at present these do not extend to integrated clinical academic training. The aim of this study was to agree consensus quality indicators for integrated clinical-academic training posts to allow benchmarking and improvement of academic surgical training in the UK.

**Appendix S3. Full research methods**

This study followed a modified nominal group consensus methodology across five sequential stages: (1) in-person scoping exercise; (2) virtual stakeholder consultation; (3) nominal group consensus meeting; (4) virtual stakeholder feedback; (5) dissemination and implementation (*Supplementary* *Figure 1*). Design and reporting followed best practice methodology for consensus research^5^.

*Stage 1: Scoping exercise*

An in-person scoping discussion around clinical academic training in surgery was held as a dedicated parallel session at the UK National Research Collaborative Meeting on 6^th^ December 2018. UK trainees and trainers with an interest in academic surgical training were invited to participate in this open-access session. There were no limitations related to training stage imposed. Four questions were posed as part of the scoping exercise:

- What do we know already?
- What works well in integrated clinical academic training and how can we measure this?
- What works poorly in integrated clinical academic training and how can we measure this?
- Who do we need to involve?

Responses were used to create a list of domains for the virtual stakeholder consultation (Stage 2).

*Stage 2: Virtual stakeholder consultation*

Following the scoping exercise, stakeholders were invited to take part in the longlisting exercise of academic Quality Indicators (QIs). Contributors were invited from the Association of Surgeons in Training mailing list (10,000 subscribers), social media (@ASiTofficial, over 8,000 followers), and via regional training programme directors through the UK Clinical Academic Training Forum. All core stakeholders had to either be an integrated clinical academic trainee in a recognised surgical specialty (past or current) or a non-integrated surgical trainee with an academic interest. All academic QIs were required to be examples of existing real-world practice in one or more Local Education and Training Boards (i.e., deemed to be feasible not aspirational). Balancing both formal integrated trainees and non-integrated trainees was determined to be essential to ensure that equitable, inclusive, and acceptable recommendations were prioritised during the consensus process. The longlist of QIs was extended and refined using an online collaborative platform (Google Docs^®^; Google Corporation, Mountain View, CA). All stakeholders were able to contribute and comments on any proposed QI. Discussion was continued on an online forum (GoogleGroups^®^; Google Corporation, Mountain View, CA) over a 4-month period from October 2018 to January 2019. At the end of Stage 2, all stakeholders were invited to participate in the in-person Nominal Group consensus meeting in Stage 3.

*Stage 3: Modified nominal group consensus*

This study adopted a modified Nominal Group Technique to derive the final set of included academic QIs^5-9^. The Nominal Group Technique is a consensus method for item generation and rapid prioritisation, ensures all voices are heard in a multi-dimensional group setting and facilitates agreement on the relative importance of problems and solutions. The Nominal Group meeting was held at the ASiT 2019 conference on 23 March 2019 in Belfast. The expertise matrix was designed to sample trainees of different sexes (male / female), training grades (postgraduate year 1-2 / year 3-5 / year 5 and above) and types (current integrated academic trainees / past integrated academic trainees / trainees with an academic interest). Silent generation was permitted to allow themes and statements to evolve from within the Nominal Group that had not previously been submitted. These statements were then added to the longlist from Stage 2 for full consideration by the group. Anonymity was maintained for authors of all submitted statements. All expert members of the nominal group were asked to submit refinements to the longlist and suggest reasons for inclusion or exclusion (a ‘round robin’)^6^. Indicator-by-indicator the session moderator (JG) covered and clarified each suggestion, with group discussion. Finally, an electronic voting phase was conducted, wherein expert members were able to:

- Accept quality indicator, with no changes to wording
- Accept quality indicator, with changes to wording
- Reject quality indicator

An acceptance rate of 80% was predefined as the minimum required for inclusion in the final quality indicator set ^10^. Nominal group members were informed of the result of each vote before moving onto discussion around the next indicator. Finally, a wider list of stakeholders was collated to contact to support dissemination and implementation in *Stage 5.*

*Stage 4: Virtual stakeholder feedback*

The agreed consensus set of quality indicators was uploaded back onto the online collaborative platform and linked back to the discussion forum for the Nominal Group (including stakeholder that could not be present for the face-to-face meeting) to review between 1^st^ October 2020 and 10^th^ December 2020. The Nominal Group reviewed final suggestions, before agreeing upon the refined quality indicator set.

*Stage 5: Dissemination and implementation*

The Nominal Group had a virtual meeting on 10^th^ December 2020 using Zoom® (Zoom Corporation, San Jose, USA) to discuss implementation of the refined quality indicator set, and pathways to impact. Examples of implementation of the ASiT integrated clinical academic surgical training quality indicators were provided by the Nominal Group and stakeholders. Key external stakeholder groups to support dissemination and implementation were also identified.

**Appendix S4. Detailed results**

*Stage 1: Scoping exercise*

Nine trainees, including 6 integrated clinical-academic surgical trainees (past or current) and 3 other non-integrated trainees with an academic interest attended the scoping exercise. The group reviewed the results of a recent survey from the ASiT clinical-academic trainee network exploring variability of experience within, and delivery of academic surgical training across the UK^4^. The group then reflected on stories and anecdotes to support these data, including positive and negative experiences of integrated clinical-academic training in surgery. Three broad domains for academic quality indicators were longlisted for review (*Supplementary Table 1*). The overall structure and objectives of the consensus process were reviewed and agreed.

*Stage 2: Virtual stakeholder consultation*

All contributors from *Stage 1* were invited to contribute to discussions in *Stage 2* of this consensus process. Seven participants continued involvement in the virtual stakeholder consultation stage. An additional 20 stakeholders joined the consultation following mailing list circulation. The list of domains was extended during the virtual stakeholder consultation, including another 4 domains (7 in total). Sixteen candidate quality indicators were drafted and refined over the consultation period for inclusion in the Nominal Group meeting.

*Stage 3: Modified nominal group consensus*

Thirteen Nominal Group members were present for the face-to-face consensus meeting with a diverse range of experiences. There were five female and eight male trainees, of whom three were postgraduate year 1-2, five were year 3-5 and five were year 5 and above, and six were current integrated academic trainees, three were past integrated academic trainees and four were trainees with an academic interest. During the nominal group consensus, the silent stage of ideas generation identified four new quality indicators that had not been previously considered. These were added to the existing longlist (N=16), with a total of 20 total quality indicators for consideration by the consensus group in the sharing ideas stage (*Table 2*). The group reviewed each quality indicator in turn in the group discussion. During round robin polling, 30.0% (6/20) of quality indicators were accepted with no change to wording, 70.0% (14/20) accepted with change to wording and 0% (0/20) rejected. After refinement, all 20 quality indicators reached the required level of agreement for inclusion in *Stage 4* virtual stakeholder feedback.

*Stage 4: Virtual stakeholder feedback*

In the final virtual stakeholder stage, wording was further refined on 7 of the 20 indicators (QI1, QI6, QI10, QI12, QI16, QI18, QI19). This improved clarity only and did not make a substantial change to the meaning or domain of the indicator. The Nominal Group and other virtual stakeholders together agreed the final indicators across seven domains:

1. Supervision and mentorship (3/20)
2. Protected academic time (3/20)
3. Clinical work (3/20)
4. Audit and accountability (2/20)
5. Financial assistance and resourcing (3/20)
6. Training progression (3/20)
7. Recruitment (1/20)

*Stage 5: Dissemination and implementation*

Key external stakeholder groups were identified as the NIHR Academy (coordinate and lead all medical integrated training programmes in the UK), the Joint Committee on Surgical Training (coordinate all surgical training programmes and curricula), Academic and Clinical Training Programme Directors (appointed in each training region of the UK), the Royal Colleges of Surgeons of England and Edinburgh, the Royal College of Physicians and Surgeons of Glasgow and the UK’s cross-funder and cross-national Clinical Academic Training Forum. Illustrative examples of how the final quality indicators are being implemented in current practice were provided by the virtual stakeholder group (*Supplementary table 3*).

The Nominal Group proposed an annual audit of the academic quality indicators with snowball sampling across the ASIT network, with results reported back at a regional level to the key external stakeholder groups to collaboratively create pathways to improve future academic training. The audit will capture experience of integrated clinical-academic trainees in surgery over their preceding 12-months of experience. The audit tool is live and available from 21^st^ September 2021 at: <https://bit.ly/ASiTAQI>.

**Appendix S5. Detailed discussion**

This modified Nominal Group consensus process has agreed and prioritised 20 quality indicators for integrated academic surgical training in the UK. The group has created an annual audit process to provide feedback to key external stakeholder groups on an annual basis. These quality indicators are expected to be the minimum provided by each deanery for integrated academic training, and should be deliverable with existing resources (i.e., not detract from resources available for non-integrated trainees) (Supplementary figure 2). This is likely to have two main benefits. First, it will empower local integrated clinical academic trainees with a set of agreed quality standards to discuss with their local training directors and hospital administrators where these are not being reached (a ‘bottom up’ approach). Second, we hope that by leveraging real-world data related to these standards in the future, we will be able to work with key external stakeholders to promote high-quality, inclusive, and enjoyable integrated academic-clinical training (a ‘top down’ approach).

As a craft specialty, surgery is likely to have some unique challenges in integrating clinical and academic work ^11^. Reaching training progression milestones in surgical specialties is already challenging in full-time clinical training ^12-15^. Moreover, trainer-trainee relationships are integral to maximising training opportunities, so lack of awareness and understanding of integrated pathways can lead to dissatisfaction for both parties ^16-18^. Integration of academic training time with clinical practice must therefore be effective and equitable in a system which both promotes and supports academic training. Whilst several quality assurance systems exist within academic training, including an infrastructure of academic training programme directors, General Medical Council survey questions (included from 2019) for academic training, and programme-exit surveys (e.g., award of competitive external fellowships, progression into senior academic posts) we hope that these high-fidelity quality indicators provide more detailed data on how integrated training is operationalised in trusts across the UK^2^. As far as we are aware this is the first trainee-led initiative to improve advocacy for optimal integration of clinical and academic training through a defined quality indicator set and annual audit process. We hope that this approach may be transferable across other medical specialties in the UK.

Whilst the formal NIHR integrated clinical academic training pathway is specific to England only, comparable programmes exist in Wales (Welsh Clinical Academic Training scheme), Northern Ireland (through the Northern Ireland Medical & Dental Training Agency) and Scotland (Scottish Clinical Research Excellence Development Scheme) so these quality indicators are likely to be applicable across all four-nations. In our implementation and dissemination phase we will promote regional adaptation to tailor the indicator set to specific local issues or challenges.

This study has several limitations. First, our sampling frame for the Nominal Group only included surgical trainees, so is likely to reflect their priorities rather than those of trainers, programme directors or funders. However, all included quality indicators represent ‘real-world’ examples of integrated training, so have been demonstrated to be feasible. The indicators therefore highlight areas of variability across regions. The expertise matrix also included both formal integrated academics and trainees with a research interest so is planned to be equitable, and acceptable to all surgical trainees (i.e., raising the bar overall, rather than unfairly advantaging a subset of trainees). Finally, by identifying and pre-planning a dissemination and implementation phase we hope to work together with key external stakeholder groups to advocate for high-quality integrated training for every academic surgical trainee. Second, we adopted a Nominal Group consensus methodology rather than other methods such as Delphi consensus. This had both strengths and weaknesses. It allows rapid and multidimensional consensus to be obtained and promotes equality and diversity of voices in ideas generation and group discussion, however it limits the capacity for convergence of ideas over time, or recognition of priorities across different subgroups of respondents^7,8^. We used a pragmatic, modified, multi-stage approach including opportunities from online comment and refinement of the quality indicators outside of the group meeting which mitigated against some of these risks. This has parallels to a nominal focus group meeting previously proposed^19^. Third, there was dropout between virtual and face-to-face rounds of the consensus process. However, sampling remained diverse within the expertise matrix at all stages ensure a breadth of voices, and asynchronous virtual editing phases allowed all stakeholders to contribute on an equal footing.

These consensus quality indicators for academic training pathways and embedded annual audit process aims to both set and monitor standards for integrated training in surgery. ASiT aims to use these data to improve retention and diversity of clinical academic trainees as future clinical research leaders to benefit both patients and the NHS.

**Collaborating author list**

Glasbey J, Blencowe N, Barclay J, Beamish AJ, Bhatia S, Bolton W, Brennan C, Chapman SJ, Clements JM, Elsey E, Fish R, Gokani VJ, Humm G, Hurst K, Hutchinson P, Jones C, Kasivisvanathan V, Keates N, Lawday S, Lee MJ, Lovegrove C, Mann H, Mason J, McDermott F, McLean K, Mohan H, Nally D, Pucher P, Staight S, Sorial AK, Trout I, Burke J.

**Appendix references**

1. Tunbridge M. Monitoring the clinician scientist scheme. *Clin Med (Lond)*. Mar-Apr 2004;4(2):141-3. doi:10.7861/clinmedicine.4-2-141

2. Clough S, Fenton J, Harris-Joseph H, et al. What impact has the NIHR Academic Clinical Fellowship (ACF) scheme had on clinical academic careers in England over the last 10 years? A retrospective study. *BMJ Open*. Jun 12 2017;7(6):e015722. doi:10.1136/bmjopen-2016-015722

3. Waqar M, Davies BM, Zakaria R, et al. Academic neurosurgery in the UK: present and future directions. *Postgrad Med J*. Oct 2019;95(1128):524-530. doi:10.1136/postgradmedj-2019-136805

4. Blencowe NS, Glasbey JC, McElnay PJ, Bhangu A, Gokani VJ, Harries RL. Integrated surgical academic training in the UK: a cross-sectional survey. *Postgrad Med J*. Apr 13 2017;doi:10.1136/postgradmedj-2016-134737

5. Waggoner J, Carline JD, Durning SJ. Is There a Consensus on Consensus Methodology? Descriptions and Recommendations for Future Consensus Research. *Acad Med*. May 2016;91(5):663-8. doi:10.1097/acm.0000000000001092

6. McMillan SS, King M, Tully MP. How to use the nominal group and Delphi techniques. *Int J Clin Pharm*. Jun 2016;38(3):655-62. doi:10.1007/s11096-016-0257-x

7. Lavallee DC, Lawrence SO, Avins AL, et al. Comparing three approaches for involving patients in research prioritization: a qualitative study of participant experiences. *Res Involv Engagem*. 2020;6:18. doi:10.1186/s40900-020-00196-4

8. Paz-Pascual C, Artieta-Pinedo I, Grandes G. Consensus on priorities in maternal education: results of Delphi and nominal group technique approaches. *BMC Pregnancy Childbirth*. Jul 24 2019;19(1):264. doi:10.1186/s12884-019-2382-8

9. Søndergaard E, Ertmann RK, Reventlow S, Lykke K. Using a modified nominal group technique to develop general practice. *BMC Fam Pract*. Jul 18 2018;19(1):117. doi:10.1186/s12875-018-0811-9

10. Prinsen CAC, Vohra S, Rose MR, et al. Core Outcome Measures in Effectiveness Trials (COMET) initiative: protocol for an international Delphi study to achieve consensus on how to select outcome measurement instruments for outcomes included in a ‘core outcome set’. OriginalPaper. *Trials*. 2014-06-25 2014;15(1):247. doi:10.1186/1745-6215-15-247

11. Pucher PH, Peckham-Cooper A, Fleming C, et al. Consensus recommendations on balancing educational opportunities and service provision in surgical training: Association of Surgeons in Training Delphi qualitative study. *Int J Surg*. Dec 2020;84:207-211. doi:10.1016/j.ijsu.2020.03.071

12. COVID-19 impact on Surgical Training and Recovery Planning (COVID-STAR) - A cross-sectional observational study. *Int J Surg*. Apr 2021;88:105903. doi:10.1016/j.ijsu.2021.105903

13. Al-Jabir A, Kerwan A, Nicola M, et al. Impact of the Coronavirus (COVID-19) pandemic on surgical practice - Part 1. *Int J Surg*. Jul 2020;79:168-179. doi:10.1016/j.ijsu.2020.05.022

14. Aziz H, James T, Remulla D, et al. Effect of COVID-19 on Surgical Training Across the United States: A National Survey of General Surgery Residents. *J Surg Educ*. Mar-Apr 2021;78(2):431-439. doi:10.1016/j.jsurg.2020.07.037

15. Early years postgraduate surgical training programmes in the UK are failing to meet national quality standards: An analysis from the ASiT/BOTA Lost Tribe prospective cohort study of 2,569 surgical trainees. *Int J Surg*. Apr 2018;52:376-382. doi:10.1016/j.ijsu.2017.09.074

16. Nally DM, Elsey E, Humm G, Mohan HM. Perceptions of the Annual Review of Competence Progression (ARCP) in surgical training in the UK and Ireland: A prospective cross sectional questionnaire study. *Int J Surg*. Jul 2019;67:117-122. doi:10.1016/j.ijsu.2018.12.009

17. Sutton PA, Beamish AJ, Rashid S, Elsey E, Mohan HM, O'Regan D. Attributes of excellent surgical trainers: An analysis of outstanding trainers. *Int J Surg*. Apr 2018;52:371-375. doi:10.1016/j.ijsu.2017.10.007

18. Whittaker JD, Davison I. A Lack of Communication and Awareness in Nontechnical Skills Training? A Qualitative Analysis of the Perceptions of Trainers and Trainees in Surgical Training. *J Surg Educ*. Jul-Aug 2020;77(4):873-888. doi:10.1016/j.jsurg.2020.01.006

19. Varga-Atkins T MJ, Willis I. Focus Group meets Nominal Group Technique: an effective combination for student evaluation. *Innovations in Education and Teaching International*. 2017;54(4)

**Table S1.** Agreed domains for virtual stakeholder consultation in *Stage 1* and *Stage 2*

| **#** | **Academic quality indicator domain** | **Stage of addition** |
| --- | --- | --- |
| **1** | Supervision and mentorship | Stage 1 |
| **2** | Protected academic time | Stage 1 |
| **3** | Clinical work | Stage 1 |
| **4** | Audit and accountability | Stage 2 |
| **5** | Financial assistance and resourcing | Stage 2 |
| **6** | Training progression | Stage 2 |
| **7** | Recruitment | Stage 2 |

| **Quality indicator** | **Longlisted indicators (Stage 2)** | **Quality indicator** | **Voting result** | **Agreed consensus indicators (Stage 3)** |
| --- | --- | --- | --- | --- |
| QI1 | Academic trainees in surgery should have an assigned academic supervisor that is matched to support development of the required skillset for academic training; where possible this should be matched to their specialty interest. | QI1 | Include, with revision | Academic trainees in surgery should have an assigned academic supervisor. Where possible this should match their specialty interest and trainees should be supported to change academic supervisors if their research interests do not match |
| QI2 | Academic trainees should set clear objectives at the start of each training year in partnership with their academic supervisors and these should be reviewed on a regular basis. | QI2 | Include, with revision | Academic supervisors should set clear objectives at the start of each training year in partnership with the academic trainees and these should be reviewed on at least a quarterly basis |
| QI3 | Academic trainees should have a dedicated named deanery academic lead whose responsibility it is to safeguard interests of the academic trainee and liaise with local trust clinicians / managers where required | QI3 | Include, with revision | Academic trainees should have a dedicated academic training program director whose responsibility it is to safeguard interests of the academic trainee and liaise with local trust clinicians / managers where required |
| QI4 | Academic trainees should have a named academic administrator who can provide information on academic training / educational opportunities and can liaise with local trusts to answer any logistical questions | QI4 | Include, with revision | Academic trainees should have a named regional academic administrator who can provide information on academic training / educational opportunities and can liaise with local trusts. |
| QI5 | Academic trainees in surgery should have protected ‘academic time’ during which are protected from clinical activities. This should be communicated to the departmental NHS managers and local clinical supervisors by the training programme director well in advance of starting the placement. | QI5 | Include, with revision | Academic trainees contracted academic time should be protected from non-academic activities. This should be communicated to the departmental NHS managers, rota coordinator and local clinical supervisors by the training programme director at least 3 months prior to starting placement, and any unmet service need covered by the trust. |
| QI6 | Academic trainees should not be required to fill ‘rota gaps’ where these arise during their dedicated academic time. | QI6 | Include | Academic trainees should not be required to fill ‘rota gaps’ where these arise during their dedicated academic time |
| N/A | - | QI7 | New, to include | If academic trainees’ clinical duties are uncovered as a result of protected academic time, the trust should be responsible for providing cover. |
| QI8 | Academic trainees should be given some 2ility to arrange their academic time in days/weeks/months according to what frequency best suits their academic work | QI8 | Include | Academic trainees should be given flexibility to arrange their academic time in days/weeks/months according to what frequency best suits their academic work |
| QI9 | Academic trainees should have key local training opportunities prioritised which may be at the expense of some service delivery. | QI9 | Include | Academic trainees should have key local training opportunities prioritised which may be at the expense of some service delivery. |
| N/A | - | QI10 | New, to include | Academic trainees that are part of an on-call rota, should be given flexibility by their hospital to reduce (or maintain) their on-call commitment as required to reflect their reduced overall clinical time. |
| QI11 | Upon a period of ‘time out’ of clinical research greater than 6 months, academic trainees should be offered a ‘back to training’ interview with their clinical and academic supervisor to ensure there are appropriately safeguarded during their initial period back in clinical work | QI11 | Include, with revision | Following an extended period of academic time, academic trainees should be able to request a ‘back to training’ interview and, where desired, keep in touch days or phased return to clinical duties. |
| QI12 | Annual audit of academic trainees’ experiences within training units should be undertaken by programme directors to ensure that trainees are not rotated into environments that are not suitable for academic trainees | QI12 | Include, with revision | Annual review of academic trainees’ experiences within training units should be undertaken by programme directors. |
| N/A | - | QI13 | New, to include | Quality indicators for academic training posts should be cleared communicated to all hospital administrative and co-ordinating staff through which academic trainees rotate. |
| QI14 | Academic trainees in surgery should have access to the same clinical study budget for additional clinical training courses as non-academic trainees. | QI14 | Include | Academic trainees in surgery should have access to the same clinical study budget for additional clinical training courses as non-academic trainees |
| QI15 | The process for academic trainees to access additional training and study budget available from the NIHR should be transparent, and made available by the named academic administrator at the start of the integrated training post | QI15 | Include, with revision | The process for academic trainees to access additional training and study budget available from the NIHR should be transparent, and made available by the academic training programme director at the start of the integrated training post |
| QI16 | Academic trainees should have a local working space, and institutional library access made available for them to complete academic work during clinical postings. | QI16 | Include | Academic trainees should have a local working space, and institutional library access made available for them to complete academic work during clinical postings. |
| QI17 | Academic trainees should have individualised clinical training plans discussed at ARCP, with flexibility and understanding in placements given on account of them having to balance academic and clinical commitments over several years | QI17 | Include, with revision | Academic trainees should have individualised clinical training plans discussed at ARCP, to provide flexibility and understanding in placements given the need to balance academic and clinical commitments. |
| QI18 | Academic trainees should have a dedicated time in their ARCP where the panel specifically review their academic objectives and progression. | QI18 | Include | Academic trainees should have a dedicated academic ARCP where they specifically review their academic objectives and outcomes. |
| QI19 | Academic trainees should have the flexibility to extend their CCT dates where required. This decision should be made with the trainee, based on competency-based progression. | QI19 | Include, with revision | Academic trainees should have the flexibility to extend their CCT dates where required, reflecting their reduced clinical commitments. This decision should be made with the trainee, based on competency-based progression. |
| N/A | - | QI20 | New, to include | Clinical training programme directors should be part of the selection panels for integrated academic clinical training posts, where possible. |

**Table S2.** Results of Nominal Group consensus meeting (Stage 3

**Table S3.** Examples of implementation of the ASiT integrated clinical academic surgical training quality indicators in practice

| **Quality**  **Indicator** | **Example of implementation** | **Trainee and region** |
| --- | --- | --- |
| QI1 | **“**In some AFP placements which are not surgically directed, foundation academic trainees interested in surgery are referred to surgical supervisors for supplementary/additional projects alongside their main project” | *NIHR Academic Clinical Fellow in General Surgery, West Midlands* |
| QI4 | “At both AFP and ACF levels, a specific named local administrator signposts academics to opportunities from the University and different hospital trusts” | *NIHR Academic Clinical Fellow in General Surgery, West Midlands* |
| QI5/QI6 | “At both AFP and ACF levels, academic time is protected, where the academic trainee is taken out of all rota and clinical environment, for day/week/months/rotations at a time, where they would not be required to fulfil any clinical commitments” | *NIHR Academic Clinical Fellow in General Surgery, West Midlands* |
| QI8 | “Trainees are supported to arrange their academic time in whatever way best meets their clinical and academic needs. Some choose regular day release; others opt to take weeks at a time while others choose three-to-six-month academic blocks” | *NIHR Academic Clinical Fellow in Urology, Oxford* |
| QI8 | “In my experience, the deanery has been supportive to alter academic time as necessary for the work at the time. Changing from blocks to weeks to days in different placements to balance against clinical work. | *NIHR Academic Clinical Fellow in ENT, Peninsula* |
| QI8 | “In my experience, this deanery is incredibly flexible as to when academic trainees take their academic time. Some prefer large blocks, others a week a month etc, and they accommodate” | NIHR Academic Clinical Fellow in Neurosurgery, Yorkshire, and The Humber |
| Q10 | “In my experience on-call commitment was always discussed and reduced as appropriate, akin to less than full time training prior to each placement” | *NIHR Academic Clinical Fellow in ENT, Peninsula* |
| QI10 | “We were contacted once we had been allocated our clinical rotations to discuss our on-call commitment and what proportion we wished to remain on the on-call rota” | *NIHR Academic Clinical Fellow in General Surgery, Severn* |
| QI11 | “For those taking time out of research, a phased return over a few weeks/keep in touch days can be organised by the deanery” | *NIHR Academic Clinical Fellow in General Surgery, West Midlands* |
| QI11 | “After a prolonged period of absence from clinical work, our deanery provides dedicated catch-up sessions via the HEE Supported Return to Training (SuppoRTT) programme” | NIHR Academic Clinical Fellow in Neurosurgery, Yorkshire, and The Humber |
| QI12 | “Our deanery has a yearly regional speciality quality panel which assesses placement and educational opportunities for academic trainees” | *NIHR Academic Clinical Fellow in Otolaryngology, Wessex* |
| QI14 | “Within the deanery there has been no barrier to my accessing a clinical study budget as in the same way as my non-academic counterparts" | *NIHR Academic Clinical Fellow in Urology, Oxford* |
| QI14 | “In this deanery, no limit is placed on academic trainees to access the clinical study budget” | *NIHR Academic Clinical Fellow in General Surgery, West Midlands* |
| QI16 | “In my deanery, academic trainees have access to a shared working space in the hospital they are working to provide a quiet and protected environment to pursue their academic work during non-clinical days. This offers opportunity to foster new collaborations and share priorities” | *NIHR Academic Clinical Fellow in General Surgery, West Midlands* |
| Q18 | “In this deanery we have a dedicated academic ARCP incorporated into our end of year ARCP, as well as regular academic reviews to ensure we are fulfilling the academic components of our training” | *NIHR Academic Clinical Fellow in General Surgery, West Midlands* |
| QI18 | “In my deanery we have an Academic representative allocated to our ARCPs to assess and advocate on our behalf.” | *NIHR Academic Clinical Fellow in ENT, Peninsula* |
| QI20 | “In my deanery the clinical and academic TPDs both sat on my interview panel. When I started my job, we each knew who the other was.” | *NIHR Academic Clinical Fellow in Urology, Oxford* |
| QI20 | “In my deanery a Clinical supervisor or TPD was on the recruitment panel for ACFs” | *NIHR Academic Clinical Fellow in ENT, Peninsula* |

**Figure S1.** Overview of study methodology

**Stage 1. Scoping exercise**
UK National Research Collaborative Meeting, Manchester, 6th December 2018

**Stage 2. Virtual stakeholder consultation**
GoogleGroups®, Online, October 2018 to January 2019

**Stage 3. Modified Nominal Group Consensus**
Association of Surgeons in Training conference, Belfast, 23rd March 2019

**Stage 4. Virtual stakeholder feedback**
GoogleGroups®, Online, October 2020 to December 2020

**Stage 5. Dissemination and implementation**
Zoom, Online, 10th December 2020

**Figure S2.** Graphical abstract presenting the final ASiT integrated clinical academic surgical training quality indicators


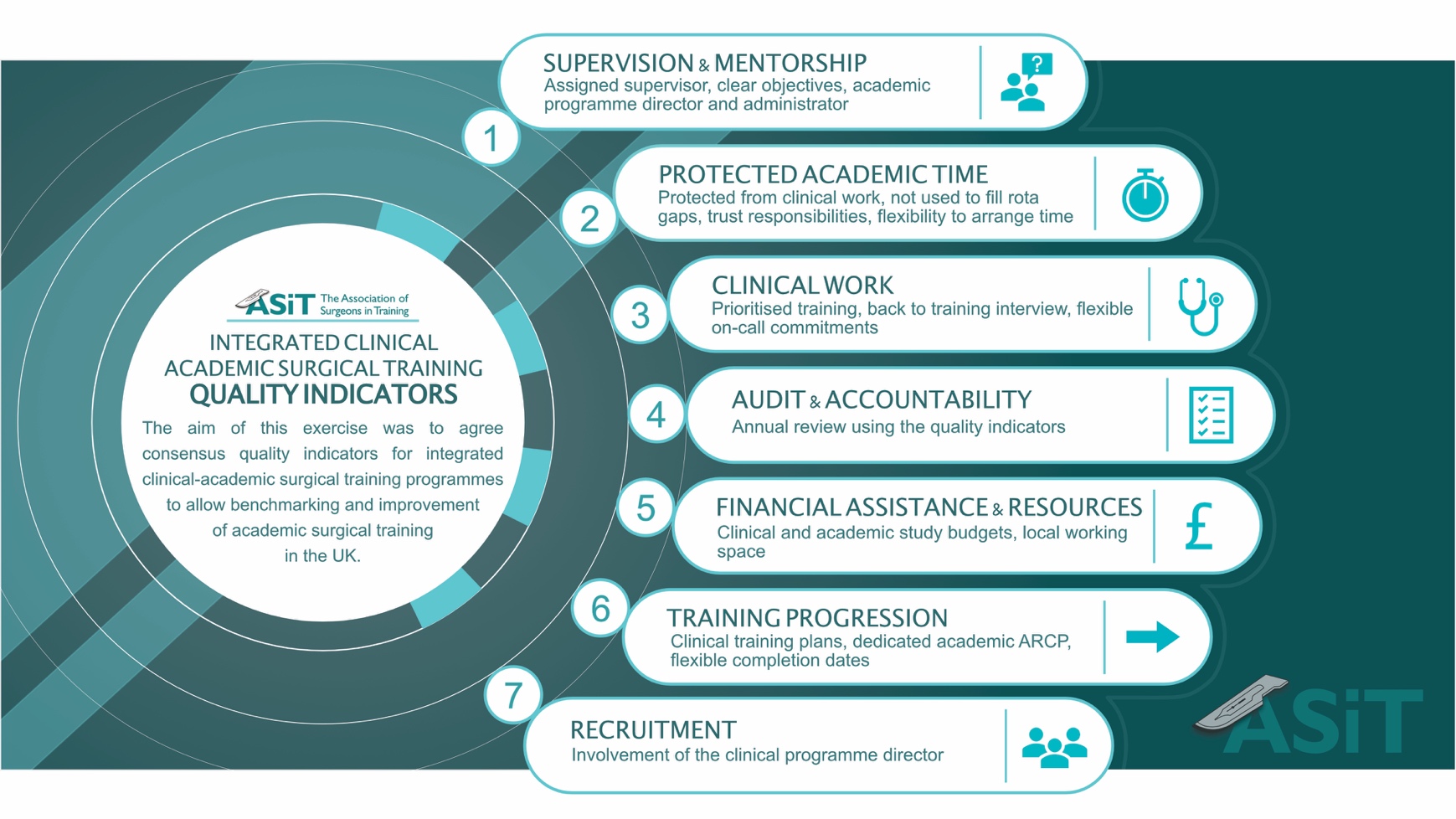

Supplement: zrac048_Supplementary_Data [file zrac048_supplementary_data.docx]
